# Genetic analysis of Japanese primary open-angle glaucoma patients and clinical characterization of risk alleles near *CDKN2B-AS1*, *SIX6* and *GAS7*

**DOI:** 10.1371/journal.pone.0186678

**Published:** 2017-12-20

**Authors:** Yukihiro Shiga, Koji M. Nishiguchi, Yosuke Kawai, Kaname Kojima, Kota Sato, Kosuke Fujita, Mai Takahashi, Kazuko Omodaka, Makoto Araie, Kenji Kashiwagi, Makoto Aihara, Takeshi Iwata, Fumihiko Mabuchi, Mitsuko Takamoto, Mineo Ozaki, Kazuhide Kawase, Nobuo Fuse, Masayuki Yamamoto, Jun Yasuda, Masao Nagasaki, Toru Nakazawa, for the Japan Glaucoma Society Omics Group (JGS-OG)

**Affiliations:** 1 Department of Ophthalmology, Tohoku University Graduate School of Medicine, Miyagi, Japan; 2 Department of Advanced Ophthalmic Medicine, Tohoku University Graduate School of Medicine, Miyagi, Japan; 3 Department of Integrative Genomics, Tohoku Medical Megabank Organization, Tohoku University, Miyagi, Japan; 4 Graduate School of Medicine, Tohoku University, Miyagi, Japan; 5 Department of Cohort Genome Information Analysis, Tohoku Medical Megabank Organization, Tohoku University, Miyagi, Japan; 6 Department of Ophthalmic imaging and information analytics, Tohoku University Graduate School of Medicine, Miyagi, Japan; 7 Department of Retinal Disease Control, Tohoku University Graduate School of Medicine, Miyagi, Japan; 8 Kanto Central Hospital of The Mutual Aid Association of Public School Teachers, Tokyo, Japan; 9 Department of Ophthalmology, Faculty of Medicine, University of Yamanashi, Yamanashi, Japan; 10 Department of Ophthalmology, University of Tokyo School of Medicine, Tokyo, Japan; 11 Division of Molecular and Cellular Biology, National Institute of Sensory Organs, National Hospital Organization Tokyo Medical Center, Tokyo, Japan; 12 Ozaki Eye Hospital, Hyuga, Miyazaki, Japan; 13 Gifu University Hospital, Gifu, Japan; 14 Medical Biochemistry, Tohoku University Graduate School of Medicine, Miyagi, Japan; 15 Graduate School of Information Sciences, Tohoku University, Miyagi, Japan; Harvard Medical School, UNITED STATES

## Abstract

**Purpose:**

To test the genetic association between Japanese patients with primary open-angle glaucoma (POAG) and the previously reported POAG susceptibility loci and to perform genotype–phenotype analysis.

**Methods:**

Genetic associations for 27 SNPs from 16 loci previously linked to POAG were assessed using genome-wide SNP data of the primary cohort (565 Japanese POAG patients and 1,104 controls). Reproducibility of the assessment was tested in 607 POAG cases and 455 controls (second cohort) with a targeted genotyping approach. For POAG-associated variants, a genotype–phenotype correlation study (additive, dominant, recessive model) was performed using the objective clinical data derived from 598 eyes of 598 POAG patients.

**Results:**

Among 27 SNPs from 16 loci previously linked to POAG, genotypes for total of 20 SNPs in 13 loci were available for targeted association study. Among 8 SNPs in 3 loci that showed at least nominal association (*P* < 5.00E-02) in the primary cohort, a representative SNP for each loci (rs2157719 for *CDKN2B-AS1*, rs33912345 for *SIX6*, and rs9913911 for *GAS7*) were selected. For these SNPs the association was found significant in both the second cohort analysis and meta-analysis. The genotype–phenotype analysis revealed significant correlations between *CDKN2B-AS1* (rs2157719) and decreased intraocular pressure (β = -6.89 mmHg, *P* = 1.70E-04; dominant model) after multiple corrections. In addition, nominal correlation was observed between *CDKN2B-AS1* (rs2157719) and optic nerve head blood flow (β = -0.54 and -0.67 arbitrary units (AU), *P* = 2.00E-02 and 1.39E-02), between *SIX6* (rs33912345) and decreased total peripapillary retinal nerve fiber layer thickness (β = -2.16 and -2.82 μm, *P* = 4.68E-02 and 2.40E-02, additive and recessive model, respectively) and increased optic nerve head blood flow (β = 0.44 AU, *P* = 2.20E-02; additive model) and between *GAS7* (rs9913911) and increased cup volume (β = 0.03 mm^3^, *P* = 4.60E-02) and mean cup depth (β = 0.03 mm^3^, *P* = 4.11E-02; additive model) and decreased pattern standard deviation (β = -0.87 dB, *P* = 2.44E-02; dominant model).

**Conclusion:**

The association between SNPs near *GAS7* and POAG was found in Japanese patients for the first time. Clinical characterization of the risk variants is an important step toward understanding the pathology of the disease and optimizing treatment of patients with POAG.

## Introduction

Glaucoma, one of the leading causes of blindness worldwide, is a neurodegenerative optic neuropathy that leads to irreversible visual field damage.[1] It is characterized by morphological changes in the optic nerve head caused by progressive loss of retinal ganglion cells (RGCs) and their axonal projections, which results in thinning of the retinal nerve fiber layer (RNFL) and enlargement of the optic nerve cup, the concave depression at the optic nerve head. As a consequence, patients have a progressive decrease in visual field sensitivity. Primary open angle glaucoma (POAG), the most common form of the disease, is considered to have multifactorial etiologies, which include elevated intraocular pressure (IOP), systemic or ocular blood flow abnormalities, older age, myopia and oxidative stress.[2–5]

Family history is also a well-known risk factor for POAG. First-degree relatives of patients have a three- to nine-fold higher risk of disease development compared with the general population.[6,7] This indicates that genetic components play important roles in the pathogenesis of POAG. Recent progress in genome-wide association studies (GWASs) has uncovered at least 16 susceptibility loci for POAG, although most investigations were performed in cohorts of Caucasian ancestry.[8–12] Several GWASs targeting POAG in Asians have been reported.[13–19] These studies have uncovered a few disease-associated loci, including those near *ABCA1*, *PMM2* and *CDC7*/*TGFBR3*.[18,19] In Japan, a few GWASs have shown variable results.[13–17] Only the susceptibility loci at 9p21 (near *CDKN2B-AS1*) and 14q23 (near *SIX6*) appear to be reproducible so far. Moreover, risk variants in 3 disease loci near *FOXC1*, *ATXN2* and *TXNRD2* recently identified [12] have never been assessed by a targeted association study and the landscape of genetic factors influencing POAG in the Japanese population remain elusive.

Despite the rapid developments in the discovery of risk loci and alleles in patients with POAG, clinical characterization of the identified loci is at a preliminary stage. This is partly because the relatively low contribution of each locus to the disease (odds ratios typically range from 1.1 to 2.0) is expected to result in subtle clinical changes unique to the disease-associated single nucleotide polymorphisms (SNPs). For this reason, genotype–phenotype correlation often requires clinical data from a large number of patients. Furthermore, due to the variability in the clinical characterization of a given patient between ophthalmologists and particularly between clinics, genotype–phenotype correlation using clinical data from different clinics is complicated. Nevertheless, multicenter studies have revealed associations between risk alleles near *CDKN2B-AS1* loci and vertical cup-to-disk ratio and decrease in IOP in Caucasian patients[20,21] and between a risk variant near *SIX6* and peripapillary RNFL thickness in European and Chinese Singaporean patients.[22–24]

The aim of this study was test for association between POAG and the disease-associated loci in the Japanese population and to assess the genotype–phenotype correlations between risk variants and clinical features of the disease.

## Materials and methods

### Study subjects

**Fig 1:**
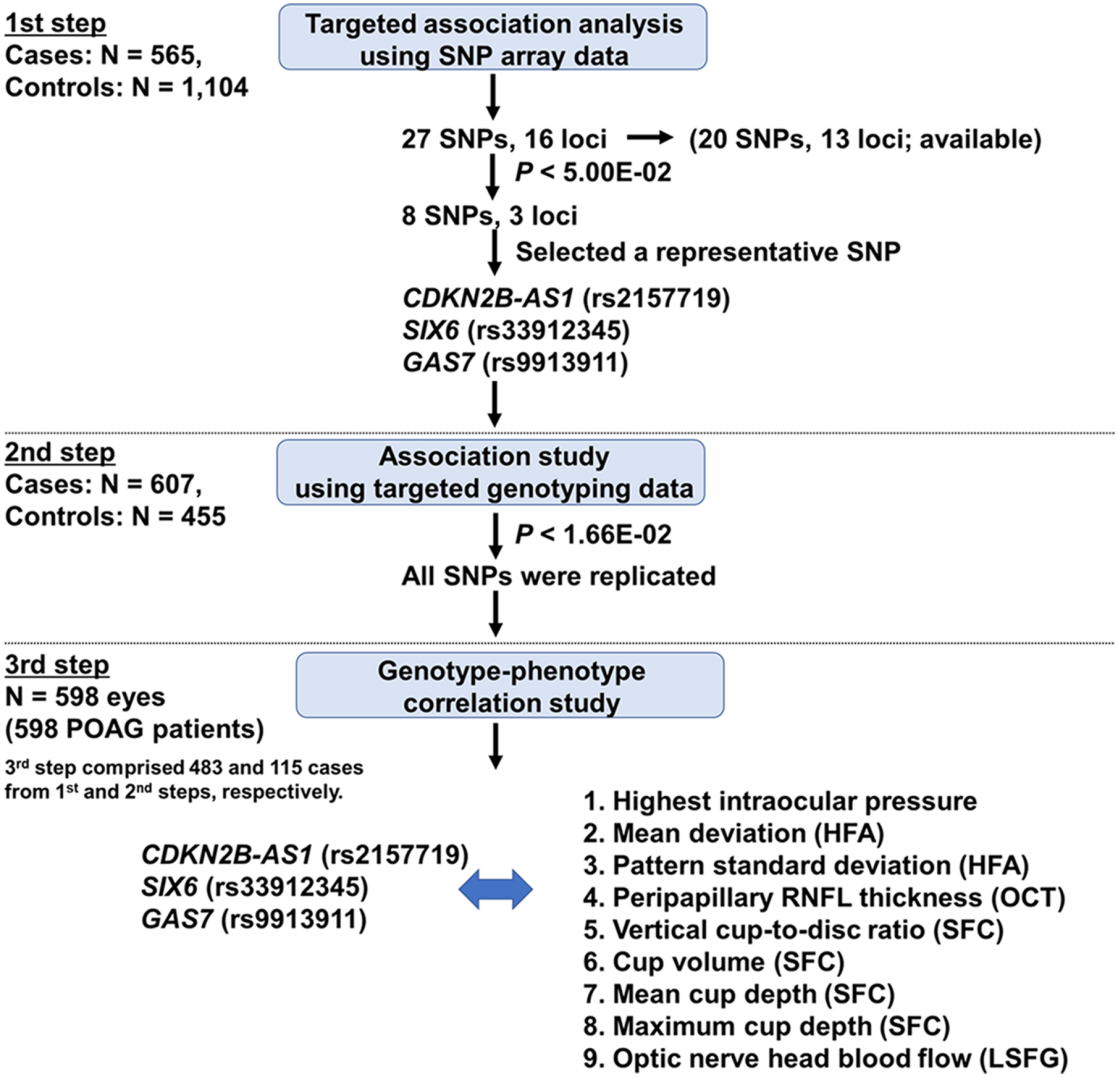
Design of the study. The study comprised 3 steps. In the first step, an association study of known POAG-related loci was carried out using the genome-wide SNP data from SNP array (cases) and a previous genetic study (control).[25–27] In the second step, 3 candidate risk SNPs were genotyped in the cases and controls to test for the reproducibility. Then a clinical correlation study was performed in the third step. SNP, single nucleotide polymorphism; POAG, primary open-angle glaucoma, HFA, Humphrey Field Analyzer, RNFL, retinal nerve fiber layer thickness, OCT, optical coherence tomography, SFC, stereoscopic fundus camera, LSFG, laser speckle flowgraphy.

The study protocol followed the tenets of the Declaration of Helsinki and was approved by the Institutional Review Board of the Tohoku Graduate School of Medicine. All participants signed a written consent form following an explanation of the nature and possible consequences of the study. All participants in this study were at the age of 35 years or older and of Japanese residents. The experimental design, which comprises three-step analyses, is outlined in Fig 1.

All of the subjects with POAG were diagnosed by glaucoma specialists and fulfilled the following diagnostic criteria: presence of glaucomatous optic disk changes, including neuroretinal rim thinning, notching, or cupping; presence of visual field defect that could be attributed to the optic disk changes; and no history of secondary, angle closure, or congenital glaucoma. For the first step, all the POAG patients were recruited at the Institutes related to Tohoku University. The control subjects recruited at the Tohoku Medical Megabank Organization as a part of prospective cohort study were considered to have no POAG based on self-report. [27] In the replication study, the POAG patients were recruited by the members of Tohoku University and the Japan Glaucoma Society Omics Group (JGS-OG), whereas the control subjects were recruited at the Institutes related to Tohoku University. The outline of the case and the control subjects are summarized in S1 Table.

### Genotyping

Japonica array (Toshiba, Tokyo, Japan) is a custom-designed array optimized for the Japanese population based on the information from the reference panel from 1,070 Japanese.[25,26] Genome-wide genotype data set was obtained using this SNP array for 602 patients with POAG according to the manufacturer’s instructions. Thirty-seven case samples that did not satisfy the quality control criteria (DishQC > 0.82 and call rates > 0.970) were excluded, thus resulting in a dataset comprising 565 cases. As a control, genotype data from 1,104 healthy subjects collected previously through a prospective cohort study [27] were used. Both cases and controls were genotyped using the same array at the same time.

SNP quality control was applied for the imputation procedure. We used info score using IMPUTE2, and considered a SNP with info score great than 0.9 as an acceptable well-imputed variant in this study. Autosomal SNPs that were assigned to ‘Recommended’ by the Ps_Classification program in the SNPolisher package (Affymetrix) were selected. We applied the following thresholds for quality control in further data cleaning: Hardy–Weinberg equilibrium with a *P* value <1.00E-04 for control samples, call rate for each SNP > 0.990, and minor allele frequencies <5.00E-02. As a result, a total of 557,352 SNPs on autosomal chromosomes passed the quality control filters and were used for whole genome imputation. Prephasing was first conducted with these SNPs by SHAPEIT (v2.r644) with options—burn 10,—prune 10, and—main 25. Genotype imputation was performed on the phased genotypes with IMPUTE2 (ver. 2.3.1) by using a phased reference panel of 1,070 healthy Japanese individuals (1KJPN panel). For IMPUTE2, the following options were used: -Ne 20000, -k_hap 1000, and -k 120.

Targeted genotyping of 3 SNPs in the second cohort was carried out by the TaqMan assay (Applied Biosystems, Carlsbad, CA, USA) and 7500 Fast Real-Time PCR systems (Applied Biosystems, Foster City, CA, USA). The call rate was over 0.990 in all of the three SNPs and was therefore considered informative.

### Clinical assessment

To determine the clinical features associated with the risk variants for the disease, standardized clinical data obtained from 598 eyes of 598 patients with POAG (patients with sufficient clinical data in the primary and second cohorts were combined) seen at Tohoku University Hospital were analyzed. The details of the clinical data extracted for the genotype–phenotype correlation study are as follows. The highest recorded IOP measured with Goldman applanation, together with central corneal thickness with the use of anterior segment optical coherence tomography (OCT) (CASIA, Tomey Cooperation, Nagoya, Japan). The mean deviation and pattern standard deviation were assessed with the 24–2 test program of a Humphrey Field Analyzer (Carl Zeiss Meditec, Dublin, CA), and the worse eye from each of the subject was used for the analysis. Peripapillary RNFL thickness was measured by OCT (3D OCT 2000, Topcon, Tokyo, Japan) together with axial length (IOLMaster, Carl Zeiss, Oberkochen, Germany). Images with image quality >60 were used for the analysis in accordance with our previous investigation. [28] To assess the morphology of the optic nerve head, data from stereoscopic fundus camera photographs (nonmyd WX, Kowa Company, Nagoya, Japan) were analyzed with built-in software. The principles of stereoscopic photography have been described in detail previously. [29] The parameters reflecting optic nerve head morphology assessed in this study included vertical cup-disk ratio, cup volume, mean cup depth, and maximum cup depth. Optic nerve head blood flow was measured with laser speckle flowgraphy (LSFG, LSFG-NAVI, Softcare Co., Fukutsu, Japan). The principles of LSFG have been described in detail previously.[30,31] We used built-in software that accompanies the LSFG-NAVI device to calculate mean blur rate (MBR), a relative index of blood flow that is expressed in arbitrary units (AU). This study focused on MBR in the tissue area, since a previous investigation had shown that this measurement can be used reliably for intergroup comparisons. [32]

### Statistical analysis

For the primary screening step, logistic regression analysis was applied to imputed SNPs with age and sex as covariates. For the analysis of selected POAG-associated SNPs, Fisher’s exact test was used to compare the frequency of each SNP between cases and controls in the replication study. The inverse variance weighted method was used for meta-analysis. We performed power calculations for POAG associations using the CaTS Genetic Power Calculator (http://www.sph.umich.edu/csg/abecasis/CaTS/index.html). In Step 2, the study had greater than 80% power to detect an association at an alpha level of 1.66E-02 (0.05 / 3) between POAG and SNPs in *CDKN2B-AS1*, *SIX6* and *GAS7* assuming an OR of 1.61, 1.42, 1.21 respectively, (extrapolated from Step 1) and additive genetic model.

Tests for heterogeneity to assess consistency across the primary and replication analyses were performed with Cochran’s Q test. An allele–dosage regression model was applied for genotype–phenotype correlation. Since genotypes for the selected three variants were all obtained with direct genotyping, we assumed additive/dominant/recessive genetic models where the dosage of the SNP was a variable ranging between 0, 1, 2/ 0, 1, 1 / 0, 0, 1 representing the number of copies of the risk allele carried (the T allele of rs2157719, C allele of rs33912345, and A allele of rs9913911). The analysis for highest recorded IOP was adjusted for age, sex, and central corneal thickness. IOP was adjusted for central corneal thickness because it can influence the readout. [33] The parameters for the Humphrey Field Analyzer, related to optic disk morphology, including vertical cup-disk ratio, cup volume, mean cup depth, and maximum cup depth, and optic nerve head blood flow were adjusted for age, sex, highest recorded IOP as previously reported.[34] Peripapillary RNFL thickness was adjusted for age, sex, and axial length.[23] The nominal significance level was set at *P* value < 5.00E-02 for each step. Bonferroni corrected *P* value was adopted taking multiple testing into consideration (*P*_corrected_). The difference was considered significant at *P*_corrected_ < 2.50E-03 (0.05/20 SNPs) for the first step of the case-control genetic analysis and the meta-analysis, *P*_corrected_ < 1.67E-02 (0.05/3 SNPs) for the second step of the case-control genetic analysis, and *P*_corrected_ < 4.27E-04 (0.05/[3SNPs × 13 covariates × 3 genetic models]) for the genotype-phenotype analysis. The data were analyzed with R version 3.2.3.

## Results

This study included a total of 1,172 patients with POAG and 1,559 healthy controls. DNA samples from the participants were collected by the members of Tohoku University Hospital, the Tohoku Medical Megabank Organization, and the JGS-OG. All participants in this study were of Japanese residents. The demographic characteristics of the study populations for the primary and the replication analysis are shown in Table 1.

**Table 1:** Demographic characteristics of the study population for the primary and replication cohorts.

		Cases	Controls
**Primary cohorts**	Genotyping	Array and imputation	Array and imputation
	N	565	1,104
	Age (yr)	64.5 ± 11.7	59.7 ± 14.1
	Age range	35–94	35–88
	% female	44.4	51.1
**Replication cohorts**	Genotyping	TaqMan assay	TaqMan assay
	N	607	455
	Age (yr)	66.3 ± 13.6	74.8 ± 7.8
	Age range	35–89	60–94
	% female	50.4	59.3
**Combined**	N	1,172	1,559

Data are expressed as mean ± standard deviation. N: number of subjects.

### Targeted analysis of previously reported POAG-associated SNPs

Initially, we focus on the targeted association study of 27 SNPs in 16 loci previously linked to POAG. All disease-associated loci were discovered originally in non-Japanese POAG cohorts, and only two of them have been convincingly reproduced in Japanese patients.[15–17] Moreover, risk variants in 3 disease loci recently identified [12] have never been assessed by a targeted association study. Among the 27 reported SNPs, the array contained 20 SNPs in 13 loci for which genotype data was available (S2 Table). Among them, data for 13 SNPs were obtained with direct genotyping and 7 SNPs were imputed at info score >0.9. Through the comparison between cases and controls, 2 SNPs in *CDKN2B-AS1* and *SIX6* loci showed significant correlation with POAG after multiple testing corrections (*P*_corrected_ < 2.50E-03). In addition, 6 SNPs in 3 loci (*CDKN2BAS-1*, *SIX6*, and *GAS7*) showed nominal association (*P* < 5.00E-02). The single SNP with the lowest *P* value in each locus, except for the *SIX6* locus, was then selected for the downstream analysis. For the *SIX6* locus, two SNPs (rs33912345 and rs10483727) had a *P* value < 5.00E-02. We selected rs33912345 (*P* = 1.67E-04) for the replication study, because rs339122345 was found to alter the protein function of *SIX6*. [35] On the other hand, rs10483727, with a lower *P* value (*P* = 1.35E-04), was located in an intergenic region between *SIX1* and *SIX6*, with an unknown effect on these genes. As a result, three SNPs in three genes (rs2157719 near *CDKN2BAS-1*, rs33912345 near *SIX6*, and rs9913911 near *GAS7*) were assessed further. These three SNPs were genotyped in independent replication cohorts comprising 607 cases and 455 controls, all from Japan. The case–control comparison found significant associations between POAG and SNPs near *CDKN2B-AS1* (*P* = 7.38E-05), *SIX6* (*P* = 7.20E-03), and *GAS7* (*P* = 1.47E-02; Table 2), taking multiple testings into consideration (*P*_corrected_ < 1.67E-02). When the data from the primary and the replication cohorts were combined and reanalyzed (Cochran’s Q test for heterogeneity, *P* = 0.701–0.871), increased significance levels were observed for SNPs near *CDKN2B-AS1* (*P* = 5.78E-09), *SIX6* (*P* = 4.33E-06) and *GAS7* (*P* = 3.32E-04), which surpassed the significance level after correcting for multiple testings (*P*_corrected_ < 2.50E-03).

**Table 2:** Summary of SNPs found to be associated with POAG by a targeted genotyping approach.

SNP ID	rs2157719	rs33912345	rs9913911
Risk allele	(T)	(C)	(A)
Nearest gene	*CDKN2B-AS1*	*SIX6*	*GAS7*
Function	intronic	missense	Intronic
Chr	9	14	17
Position (hg19, bp)	22,033,366	60,976,537	10,031,183
**Primary cohorts**			
Frequency	0.893/0.838	0.832/0.772	0.443/0.396
*P*	**1.42E-05**	**1.67E-04**	9.33E-03
OR (95% CI)	1.61 (1.29–2.00)	1.42 (1.18–1.71)	1.21 (1.05–1.40)
**Replication cohorts**			
Frequency	0.888/0.828	0.820/0.772	0.449/0.395
*P*	**7.38E-05**	**7.20E-03**	**1.47E-02**
OR (95% CI)	1.65 (1.28–2.14)	1.34 (1.08–1.67)	1.24 (1.04–1.48)
**Meta-analysis**			
*P*	**5.78E-09**	**4.33E-06**	**3.32E-04**
OR (95% CI)	1.63 (1.38–1.92)	1.38 (1.20–1.59)	1.22 (1.09–1.37)
*P*_het_	0.871	0.701	0.828

Significance level was set at *P*_corrected_ < 2.50E-03 (0.05/20 SNPs) for the first step and the meta-analysis and at *P*_*corrected*_ < 1.67E-02 (0.05/3 SNPs) for the second step after Bonferroni correction. Bold texts indicate values with a statistically significant difference after the correction. Chr, chromosome; bp, base pair; Frequency, frequency of risk alleles for each case and control; OR, odds ratio; CI, confidence interval; *P*_het_, *P* value of heterogeneity by Cochran’s Q test.

### Clinical characterization of risk variants near *CDKN2B-AS1*, *SIX6* and *GAS7*

Next, a genotype–phenotype correlation study was performed to determine the clinical features associated with the risk variants near *CDKN2B-AS1*, *SIX6* and *GAS7* that were found to be correlated with POAG in Japanese patients. Clinical data from 598 eyes of 598 patients with POAG seen at Tohoku University Hospital (from both the primary and the replication cohorts), which were obtained in identical clinical settings, were analyzed. The clinical background of the patients and the profile of the parameters assessed are summarized in Table 3. Nine quantitative traits were selected for the genotype–phenotype correlation analysis and the multivariate regression model was applied. These included the highest recorded IOP, parameters reflecting visual field sensitivity measured with the Humphrey Field Analyzer (mean deviation and pattern standard deviation), RNFL thickness obtained by OCT, parameters of optic disk morphology measured by stereoscopic fundus camera and optic nerve head blood flow measured by LSFG. The results of the correlation analysis are presented in Table 4.

**Table 3:** Clinical demographics of patients in the genotype–phenotype correlation study.

Age (yr)	64.1 ± 11.7
Sex (male:female)	264:334
Axial length (mm)	25.2 ± 1.7
Central corneal thickness (μm)	512 ± 36
Highest recorded IOP (mm Hg)	18.3 ± 5.9
Humphrey Field Analyzer parameters	
: mean deviation (dB)	−13.4 ± 8.63
: pattern standard deviation (dB)	9.62 ± 3.78
Peripapillary RNFL thickness	
: total (μm)	79.3 ± 14.0
: superior (μm)	90.2 ± 21.6
: temporal (μm)	70.4 ± 17.0
: inferior (μm)	81.2 ± 20.9
: nasal (μm)	75.1 ± 15.7
Vertical cup-to-disk ratio	0.842 ± 0.075
Cup volume (mm^3^)	0.340 ± 0.261
Mean cup depth (mm)	0.231 ± 0.238
Maximum cup depth (mm)	0.592 ± 0.535
Optic nerve head blood flow (AU)	9.00 ± 2.37

AU, arbitrary units; IOP, intraocular pressure; RNFL, retinal nerve fiber layer.

**Table 4:** Association between genetic variants and clinical parameters.

	**rs2157719 near *CDKN2B-AS1* (N = 598)**		
	**Additive model**	**Dominant model**	**Recessive model**
Variable	*P* value	*P* value	*P* value
Highest recorded IOP (N = 597)	5.77E-03 (-1.59 ± 0.57)*	1.70E-04 (-6.89±1.82)*	0.06
HFA parameters			
: mean deviation (N = 597)	0.95	0.75	0.96
: pattern standard deviation (N = 597)	0.42	0.95	0.35
Peripapillary RNFL thickness			
: total (N = 574)	0.17	0.29	0.23
: superior (N = 574)	0.13	0.85	0.09
: temporal (N = 574)	0.08	0.23	0.12
: inferior (N = 574)	0.47	0.32	0.62
: nasal (N = 574)	0.95	0.37	0.71
Vertical cup-to-disk ratio (N = 451)	0.66	0.54	0.76
Cup volume (N = 451)	0.95	0.84	0.89
Mean cup depth (N = 451)	0.36	0.69	0.37
Maximum cup depth (N = 451)	0.30	0.44	0.36
ONH blood flow (N = 503)	2.00E-02 (-0.54±0.23)*	0.60	1.39E-02 (-0.67±0.27)*
	**rs33912345 near *SIX6* (N = 596)**		
	**Additive model**	**Dominant model**	**Recessive model**
Variable	*P* value	*P* value	*P* value
Highest recorded IOP (N = 595)	0.94	0.82	0.99
HFA parameters			
: mean deviation (N = 595)	0.30	0.82	0.27
: pattern standard deviation (N = 595)	0.17	0.47	0.19
Peripapillary RNFL thickness			
: total (N = 572)	4.68E-02 (-2.16± 1.08)*	0.93	2.40E-02 (-2.82±1.24)*
: superior (N = 572)	1.33E-02 (-4.11± 1.65)*	0.28	1.40E-02 (-4.69±1.90)*
: temporal (N = 572)	0.87	0.05	0.37
: inferior (N = 572)	0.06	0.85	3.91E-02 (-3.88±1.87)*
: nasal (N = 572)	0.28	0.47	0.33
Vertical cup-to-disk ratio (N = 449)	0.70	0.83	0.72
Cup volume (N = 449)	0.42	0.81	0.41
Mean cup depth (N = 449)	0.16	0.24	0.24
Maximum cup depth (N = 449)	0.24	0.37	0.30
ONH blood flow (N = 501)	2.20E-02 (0.44 ± 0.19)*	0.05	0.05
	**rs9913911 near *GAS7* (N = 596)**		
	**Additive model**	**Dominant model**	**Recessive model**
Variable	*P* value	*P* value	*P* value
Highest recorded IOP (N = 595)	0.86	0.94	0.75
HFA parameters			
: mean deviation (N = 595)	0.10	0.05	0.41
: pattern standard deviation (N = 595)	0.24	2.44E-02 (-0.87±0.38)*	0.88
Peripapillary RNFL thickness			
: total (N = 572)	0.91	0.82	0.98
: superior (N = 572)	0.83	0.70	0.99
: temporal (N = 572)	0.56	0.57	0.69
: inferior (N = 572)	0.76	0.79	0.81
: nasal (N = 572)	0.63	0.29	0.87
Vertical cup-to-disk ratio (N = 449)	0.90	0.94	0.80
Cup volume (N = 449)	4.60E-02 (0.03±0.01)*	0.08	0.12
Mean cup depth (N = 449)	4.11E-02 (0.03±0.01)*	0.07	0.10
Maximum cup depth (N = 449)	0.13	0.25	0.18
ONH blood flow (N = 501)	0.57	0.10	0.59

Significance level was set at *P*_corrected_ < 4.27E-04 (0.05/[3 SNPs × 13 covariates × 3 genetic models]) for the genotype-phenotype analysis after Bonferroni correction. Bold texts indicate values with a statistically significant difference after the correction.

* indicates *P* value (β±SE)

β, changes in clinical parameters per copy of the risk allele; SE, standard error,

N, The numbers of eyes used for each variable and the risk allele for each SNP were displayed. IOP, intraocular pressure. HFA, Humphrey Field Analyzer. RNFL, retinal nerve fiber layer. ONH, Optic nerve head.

The presence of each POAG risk allele near *CDKN2BAS-1* (rs2157719) was associated with a decrease in IOP of 6.89 mmHg (*P* = 1.70E-04; dominant model). No association was found between the other two variants and IOP. The presence of the risk allele in rs33912345 near SIX6 was nominally associated with a decrease in total peripapillary RNFL thickness of 2.16 μm (*P* = 4.68E-02; additive) and 2.82 μm (*P* = 2.40E-02; recessive). When the peripapillary RNFL was further divided into superior, inferior, nasal and temporal quadrants and assessed separately, the risk allele was nominally associated with a decrease in thickness of 4.11 μm (*P* = 1.33E-02; additive) and 4.69 μm (*P* = 1.40E-02; recessive) superiorly and 3.88 μm inferiorly (*P* = 3.91E-02; recessive), but not in other quadrants. A nominal association between parameters reflecting optic nerve head morphology and the risk allele near *GAS7* (rs9913911) was observed. Each risk allele near *GAS7* was possibly linked to an increase in cup volume of 3.00E-02 mm^3^ (*P* = 4.60E-02; additive) and mean cup depth of 3.00E-02 mm^3^ (*P* = 4.11E-02; additive). The presence of the risk allele in rs2157719 near *CDKN2B-AS1* was nominally associated with a decrease in optic nerve head blood flow of 0.54 AU (*P* = 2.00E-02; additive) and 0.67 AU (*P* = 1.39E-02; recessive). The risk allele in rs33912345 near *SIX6* was nominally associated with an increase in optic nerve head blood flow of 0.44 AU (*P* = 2.20E-02; additive). The risk allele in rs9913911 near GAS7 was nominally associated with a decrease in patter standard deviation of visual field parameters of 0.87 decibels (dB) (*P* = 2.44E-02; dominant). No associations were found between visual field parameters and the other two risk variants.

## Discussion

By targeted analysis of a selection of reported risk SNPs in 1,172 Japanese patients with POAG and 1,559 ethnically matched controls, variants near *CDKN2B-AS1* (rs2157719), *SIX6* (rs33912345) and *GAS7* (rs9913911) were found to be associated with the disease in the population. The association between SNPs near *GAS7* and POAG was found in Japanese patients for the first time. This is only the third locus that has been associated convincingly with the disease in Japanese. The other two loci that were previously shown to be associated with the disease are *CDKN2B-AS1* and *SIX6* loci and both associations were confirmed in the present study. Genotype–phenotype correlation using a clinical data set derived from a single institution enabled a reliable comparison of the data from the patients and the risk variants. The results showed a unique pattern of clinical correlations for each of the risk variants in these three genes, which implies different roles of the risk genes in the development of POAG.

The results show that the disease risk variant rs2157719 near *CDKN2B-AS1* was associated with decreased IOP in Japanese patients with POAG. This counterintuitive finding that an SNP linked to a lower IOP is also a risk factor for POAG, which is exacerbated by higher IOP has been reported previously in Caucasian populations.[20,21] Nevertheless, this study confirms the seemingly confusing finding in a far eastern Asian population and also assures the validity of our analysis pipeline. There are at least 3 possible explanations for the association between rs2157719 near *CDKN2B-AS1* and a decrease in IOP. First, the risk alleles may confer the RGCs to become more sensitive to IOP-related glaucomatous damage. Second, the risk alleles may contribute to POAG through IOP-independent mechanisms. Lastly, although unlikely, lower IOP may be a disease risk for a subset of POAG patients with the risk alleles.

To our surprise, our study had another novel counterintuitive result. In the present analysis, the POAG risk SNP near *CDKN2B-AS1* was nominally associated with decreased optic nerve head blood flow, whereas the risk SNP near *SIX6* showed the inverse relationship, i.e. increased optic nerve head blood flow. On the other hand, the risk SNP near *GAS7* had no association with blood flow. Reduced optic nerve head blood flow, as measured by LSFG, has been observed in patients with glaucoma and in experimental monkey models of glaucoma by us and others.[36–43] We previously reported that optic nerve head blood flow was already reduced in preperimetric glaucoma, [44] the earliest stage of glaucoma. The blood flow further declines with increased disease severity.[37,42,43] These investigations suggest that microvascular alterations in the optic nerve head may play an important role in the pathogenesis of POAG. Although the mechanisms by which different loci influence the development of glaucoma are uncertain, there are at least two potential reasons to account for the conflicting results for the association between optic nerve head blood flow and the risk alleles in *CDKN2BAS-1* and *SIX6*. First, the risk alleles may confer the RGCs to become more sensitive to reduced ocular circulation. Second, the risk alleles may contribute to POAG through mechanisms independent of ocular circulation. Our results further highlight the complex nature of POAG, which may underlie the differences in response to treatment among patients. Our study also found that the risk allele near *SIX6* (rs33912345) was nominally associated with reduced total peripapillary RNFL thickness and also with the thickness of superior and the inferior sectors in patients with POAG, a finding that was not observed for other risk variants. The findings are also in agreement with a study conducted in Singapore that found an association between the risk allele near *SIX6* and reduced RNFL thickness in a cohort comprising 2,129 eyes from 1,222 subjects without glaucoma and 21 patients with glaucoma.[23] Therefore, the results of the present study are in good agreement with previously reported findings. Nevertheless, our study confirms the genotype–phenotype correlation in patients with glaucoma who actually suffer from the consequences of reduction in RNFL thickness. This finding greatly expands on the earlier study, in which the data were derived almost exclusively from normal subjects. The risk allele near *GAS7*, where the locus has been associated with IOP in previous studies [45,46], was found to be nominally associated with an increased cup volume and mean cup depth, as quantified from the stereoscopic fundus camera photographs but not with IOP.

The different findings for two different risk genes (*SIX6* and *GAS7*), derived from two different imaging modalities (OCT and stereoscopic fundus camera), may imply that they have different roles in their contribution to the disease. Peripapillary RNFL thickness measured by OCT presumably reflects the number of axons projecting from the RGCs. Parameters related to optic disk morphology measured by the stereoscopic fundus camera probably reflect both the number of axons projecting from the RGCs and factors related to the extracellular matrix constituting the optic disk and the lamina cribrosa. The finding of reduced peripapillary RFNL thickness in patients with the *SIX6* risk variant, but not in those with the *GAS7* variant, implies that the pathologic changes in glaucoma associated with the *SIX6* variant may occur primarily in the RGCs and their axons. The increased cupping with relatively preserved RNFL thickness observed in patients with the *GAS7* variant implies that the primary pathologic focus may reside in the disk extracellular matrix and that axonal injury and RGC death are secondary events. Koolwijk et al., using quantitative real-time polymerase chain reaction (PCR), reported that the highest expression of *GAS7* mRNA in human ocular tissues was in the lamina cribrosa, [47] a result consistent with our speculation. This suggestion could be tested further by comparing the clinical parameters in a large number of patients in the early phase of the disease before both the axons and the extracellular matrix of the optic disk degenerate in the later phase of the disease, or, alternatively, in healthy subjects.

The present study was limited by its sample size and cross-sectional design. In addition, IOP was measured in patients with POAG without discontinuing their antiglaucoma medications. The use of topical medication likely had an impact on IOP. A longitudinal study that includes a larger number of patients with POAG with complete information on pretreatment IOP is desired.

In conclusion, comprehensive SNP analysis of the known POAG-related loci in Japanese patients with POAG identified disease risk variants near *CDKN2BAS-1* (rs2157719), *SIX6* (rs33912345) and *GAS7* (rs9913911). The genotype–phenotype correlation study suggested that the risk variants in these three genes have different effects on the glaucoma phenotype. Studying the associations between risk variants and clinical parameters is an important step toward understanding the pathology of the disease and optimizing the treatment of patients with POAG.

## Supporting information

S1 Table Definitions of cases and controls. + The case or control definition criterion was applied;—the criterion was not applied.
(DOCX)

S2 Table Summary of reported POAG-associated SNPs. *P* values from logistic regression analysis adjusted for age and sex in the GWAS are displayed (far right). Genotype method for primary screening is displayed. If the SNP is imputed, info score is displayed. NA indicates that the SNP is not included in the custom chip or was not polymorphic. Chr, chromosome, SNP, single nucleotide polymorphism; bp, base pair.
(DOCX)

S1 File Dataset. (XLSX)
