# Supplementary material for: Genetic analysis of Japanese primary open-angle glaucoma patients and clinical characterization of risk alleles near CDKN2B-AS1, SIX6 and GAS7
Source: PLoS One. 2017 Dec 20;12(12):e0186678. doi: 10.1371/journal.pone.0186678 (PMC5737967; doi:10.1371/journal.pone.0186678)
Supplement: S1 Table — + The case or control definition criterion was applied;—the criterion was not applied. (DOCX) [file pone.0186678.s001.docx]

**Supporting Information**

**S1 Table. Definitions of cases and controls.**

|  | **Primary**  **cohorts** | **Replication cohorts** |
| --- | --- | --- |
| **Case definition** |  |  |
| Glaucomatous optic disk change | + | + |
| Reliable visual field defect | + | + |
| Exclusion of secondary cause of the optic head damage | + | + |
| Exclusion of occluded and closed angle | + | + |
| Exclusion of congenital glaucoma | + | + |
| **Control definition** |  |  |
| Vertical cup-to-disk ratio < 0.7 | - | + |
| Intraocular pressure < 21 mm Hg | - | + |
| No medical history of glaucoma | - | + |

+ The case or control definition criterion was applied; – the criterion was not applied.
