# Supplementary material for: Genetic analysis of Japanese primary open-angle glaucoma patients and clinical characterization of risk alleles near CDKN2B-AS1, SIX6 and GAS7
Source: PLoS One. 2017 Dec 20;12(12):e0186678. doi: 10.1371/journal.pone.0186678 (PMC5737967; doi:10.1371/journal.pone.0186678)
Supplement: S2 Table — P values from logistic regression analysis adjusted for age and sex in the GWAS are displayed (far right). Genotype method for primary screening is displayed. If the SNP is imputed, info score is displayed. NA indicates that the SNP is not included in the custom chip or was not polymorphic. Chr, chromosome, SNP, single nucleotide polymorphism; bp, base pair. (DOCX) [file pone.0186678.s002.docx]

**S2 Table. Summary of reported POAG-associated SNPs.**

| Nearest gene | Chr | SNP ID | Position (bp) | Japonica array | Rep | *P* value |
| --- | --- | --- | --- | --- | --- | --- |
| *CDC7 / TGFBR3* | 1 | rs1192415 | 920,770,97 | Direct genotyping |  | 0.206 |
| *TMCO1* | 1 | rs4656461 | 163,953,829 | Direct genotyping |  | *NA* |
|  |  | rs7555523 | 165,718,979 | Imputation (0.0) |  | *NA* |
| *FNDC3B* | 3 | rs6445055 | 171,992,387 | Direct genotyping |  | 0.414 |
| *AFAP1* | 4 | rs4619890 | 7,853,160 | Imputation (0.999) |  | 0.179 |
|  |  | rs11732100 | 7,924,690 | No data |  | *NA* |
| *GMDS* | 6 | rs11969985 | 192,207 | Imputation (0.988) |  | 0.113 |
| *FOXC1* | 6 | rs2745572 | 1,548,369 | Imputation (0.996) |  | 0.083 |
| *CAV1 / CAV2* | 7 | rs10258482 | 116,150,095 | No data |  | *NA* |
|  |  | rs4236601 | 116,162,729 | No data |  | *NA* |
| *Intergenic region* | 8 | rs284489 | 105,958,020 | Direct genotyping |  | 0.183 |
| ***CDKN2B-AS1*** | 9 | rs1063192 | 22,003,367 | Direct genotyping |  | 1.28E-03 |
|  |  | rs523096 | 22,019,129 | Direct genotyping |  | 4.46E-05 |
|  |  | rs7865618 | 22,021,005 | Direct genotyping |  | 1.76E-05 |
|  |  | **rs2157719** | 22,033,366 | Direct genotyping | YES | 1.42E-05 |
|  |  | rs7866783 | 22,056,359 | No data |  | *NA* |
|  |  | rs4977756 | 22,068,652 | Direct genotyping |  | 0.034 |
| *ABCA1* | 9 | rs2472493 | 107,695,848 | Imputation (0.992) |  | 0.190 |
|  |  | rs2487032 | 107,703,934 | Direct genotyping |  | 0.200 |
| *ABO* | 9 | rs8176743 | 136,131,415 | Imputation (0.998) |  | 0.343 |
| *ATXN2* | 12 | rs7137828 | 111,932,550 | Imputation (0.0) |  | *NA* |
| ***SIX6*** | 14 | **rs33912345** | 60,976,537 | Direct genotyping | YES | 1.67E-04 |
|  |  | rs10483727 | 61,072,875 | Direct genotyping |  | 1.35E-04 |
| *PMM2* | 16 | rs3785176 | 8,896,931 | Direct genotyping |  | 0.781 |
| ***GAS7*** | 17 | rs9897123 | 10,020,501 | Imputation (0.989) |  | 0.250 |
|  |  | **rs9913911** | 10,031,183 | Direct genotyping | YES | 9.33E-03 |
| *TXNRD2* | 22 | rs35934224 | 19,872,645 | Imputation (0.963) |  | 0.723 |

*P* values from logistic regression analysis adjusted for age and sex in the GWAS are displayed (far right). Genotype method for primary screening is displayed. If the SNP is imputed, info score is displayed. NA indicates that the SNP is not included in the custom chip or was not polymorphic. Chr, chromosome, SNP, single nucleotide polymorphism; bp, base pair.
